# A Two‐Week Treatment with Plant Extracts Changes Gut Microbiota, Caecum Metabolome, and Markers of Lipid Metabolism in ob/ob Mice

**DOI:** 10.1002/mnfr.201900403

**Published:** 2019-06-25

**Authors:** Amandine Brochot, Vincent Azalbert, Jean‐François Landrier, Franck Tourniaire, Matteo Serino

**Affiliations:** ^1^ Groupe PiLeJe 75015 Paris France; ^2^ Institut National de la Santé et de la Recherche Médicale (INSERM) Toulouse France; ^3^ Unité Mixte de Recherche (UMR) 1048 Institut de Maladies Métaboliques et Cardiovasculaires (I2MC) Université Paul Sabatier (UPS) Toulouse 31432 France; ^4^ Centre de recherche CardioVasculaire et Nutrition (C2VN) Aix‐Marseille Université, INRA, INSERM Marseille 13385 France; ^5^ CriBioM Criblage Biologique Marseille Faculté de Médecine de la Timone Marseille France; ^6^ IRSD Université de Toulouse, INSERM, INRA, ENVT, UPS Toulouse 31024 France

**Keywords:** gut microbiota dysbiosis, metabolic diseases, metabolomics, plant extracts, prebiotics

## Abstract

**Scope:**

Targeting gut microbiota dysbiosis by prebiotics is effective, though side effects such as abdominal bloating and flatulence may arise following high prebiotic consumption over weeks. The aim is therefore to optimize the current protocol for prebiotic use.

**Methods and results:**

To examine the prebiotic properties of plant extracts, two independent studies are conducted in ob/ob mice, over two weeks. In the first study, *Porphyra umbilicalis* and *Melissa officinalis L*. extracts are evaluated; in the second study, a high vs low dose of an *Emblica officinalis* Gaertn extract is assessed. These plant extracts affect gut microbiota, caecum metabolome, and induce a significant lower plasma triacylglycerols (TG) following treatment with *P. umbilicalis* and significantly higher plasma free fatty acids (FFA) following treatment with the low‐dose of *E. officinalis* Gaertn. Glucose‐ and insulin‐tolerance are not affected but white adipose tissue and liver gene expression are modified. In the first study, *IL‐6* hepatic gene expression is significantly (adjusted *p* = 0.0015) and positively (*r* = 0.80) correlated with the bacterial order Clostridiales in all mice.

**Conclusion:**

The data show that a two‐week treatment with plant extracts affects the dysbiotic gut microbiota and changes both caecum metabolome and markers of lipid metabolism in ob/ob mice.

## 1 Introduction

The gut microbiota is recognized as a major actor in host pathophysiology, with functions extending beyond those related to digestion.^1^ Both taxonomic (relative (%) abundance of bacterial groups) and functional (microbial metabolic pathways) alterations of gut microbiota, named *dysbioses*, have been associated with several pathologies, in particular metabolic diseases such as type 2 diabetes and obesity.^2^, ^3^, ^4^ Thus, multiple strategies targeting gut microbiota dysbiosis may be effective in restoring physiological conditions. One of these strategies is the use of prebiotics, originally defined as to “non‐digestible food ingredient that beneficially affects the host by selectively stimulating the growth and/or activity of one or a limited number of bacteria already resident in the colon.”^5^ In several experimental models of dysbiotic gut microbiota, including those of obesity induced by a fat‐rich diet, prebiotics dampened the effects of the diet and intestinal inflammation by acting on the gut microbiota.^6^

Nevertheless, beyond being merely beneficial, prebiotics may also induce side‐effects such as gut ballooning and/or increased flatulence.^7^, ^8^ These effects are related to both an excessive production of gas (via fermentation of prebiotics by gut bacteria^9^) and to the important amount (grams per day per patient) of prebiotics sometimes needed to achieve beneficial effects on health.

In the interest of alleviating these undesirable effects, increasing attention has been paid to the potentially prebiotic effects of substrates other than oligosaccharides. The International Scientific Association for Probiotics and Prebiotics (ISAPP) proposed the following update of the prebiotic definition: “a substrate selectively utilized by host microorganisms conferring a health benefit.”^10^ This new definition expands the concept of prebiotic to polyunsaturated fatty acids, phytochemicals, and phenolics. Recent studies have suggested that plant extracts rich in polyphenols might modulate a dysbiotic gut microbiota in various animal models including mice fed on a fat‐rich diet.^6^, ^11^ Based on this evidence, we conducted a proof‐of‐concept study in ob/ob mice, a well‐known model of gut microbiota dysbiosis (increased Firmicutes to Bacteroidetes ratio) and metabolic disease^12^ to evaluate the ability of *Porphyra umbilicalis* and *Melissa officinalis L*. extracts to induce changes in a dysbiotic gut microbiota and also to determine whether these changes may be associated with some ameliorations in both lipid and glucose metabolism.^12^ It is noteworthy that in clinical practice prebiotics are proposed to patients suffering from gastrointestinal problems possibly due to a gut microbiota dysbiosis. Thus, a good indicator for treatment by a particular prebiotic is its capacity to change an already dysbiotic gut microbiota. *Porphyra umbilicalis* is a potential functional food with a high content of dietary fibers, minerals, and trace elements as well as proteins and lipids that can exert multiple biological activities and has a high antioxidant capacity.^13^ *Melissa officinalis L*. is known to have multiple pharmacological effects including anxiolytic, antiviral, and antispasmodic activities, as well as to have an impact on mood, memory, and cognition.^14^

Then, we performed a second independent study in which ob/ob mice were treated with two doses (high vs low) of an extract of *Emblica officinalis* Gaertn (also known as amla or *Phyllanthus emblica* Linn). The fruit of *E. officinalis* Gaertn is a rich source of vitamin C and tannins and has a strong antioxidant activity.^15^, ^16^ It has also been shown to improve glucose and lipid metabolism in both normal subjects and type 2 diabetic patients, though at doses as high as two to three grams per day for three weeks.^17^ Gut (fecal) microbiota was analyzed both at baseline (before treatment) and after two weeks of treatment. The overall caecum metabolome, various markers of lipid and glucose metabolism as well as the expression of key metabolic and inflammatory genes in white adipose tissue and liver were also studied.

## 2 Experimental Section

### 2.1 Animal Model and Tissue Collection

The two independent studies described above were conducted in 12‐week‐old C57Bl/6J male ob/ob mice (Charles River, L'Arbresle, France) fed on a normal chow (NC) and then treated with plant extracts dissolved in sterile water or sterile water only (control group), as described below. Mice were group‐housed (five mice per cage) in a specific pathogen‐free controlled environment (12‐h daylight‐cycle, light off at 7:00 p.m.). At the end of the study, mice were sacrificed by cervical dislocation in a fed state (at morning) to avoid a fasting‐induced change in the composition of the gut microbiota and tissues were collected and snap‐frozen in liquid nitrogen. All experimental procedures involving animals were approved by the local ethical committee and performed in accordance with relevant guidelines and regulations (APAFIS#8111‐2016120716262061 v10).

### 2.2 Plant Extract Preparation and Composition

*Porphyra umbilicalis* thallus was collected in France (Brittany) in 2013. The extract of *Porphyra umbilicalis* (PiLeJe Industrie, France) is made by extraction of 1 part of dry *P. umbilicalis* in 20 parts of 75% ethanol at 40 °C during 4 h. After settling, the solid part is dried under reduced pressure and sieved for packaging (native extract ratio [NER]: 0.65 to 0.75: 1; drug extract ratio [DER]: 0.65 to 0.75: 1).

*Melissa officinalis L*. leaves were collected in France (Auvergne) in 2015. The extract of *M. officinalis L*. tested (Lemon balm ipowder PiLeJe Industrie, France; Dubourdeaux M (2009), Thiomed Saint Bonnet de Rochefort EP2080436A2, https://patentimages.storage.googleapis.com/c6/89/1e/07a71ba38bd0d5/EP2080436A2.pdf) is obtained by extraction of 1 part of *M. officinalis L*. ground dried leaves in 10 parts of water at 85 °C for 30 min. The resulting extract is concentrated under vacuum ([NER]: 5 to 7: 1), then fixed and dried on one part of *M. officinalis L*. ground dried leaves (impregnation support) under reduced pressure (DER: 2 to 4: 1). The resulting plant extract is crushed so that it can be incorporated into capsules or tablets. An identical extraction process was used for *Emblica officinalis* Gaertn fresh fruits collected in India in 2015 (Amla ipowder PiLeJe Industrie, France; Dubourdeaux (2009); NER: 14 to 17: 1; DER: 1 to 3: 1).

Nutritional analysis of the three plant extracts (Capinov SAS, Landerneau, France; **Table** 1) showed that the extract of *P. umbilicalis* contained a high concentration of proteins and fibers compared to the extracts of *M. officinalis L*. and *E. officinalis* Gaertn. The extract of *E. officinalis* Gaertn had the highest content in carbohydrates (15 times more carbohydrates in *E. officinalis* Gaertn extract than that of *P. umbilicalis*). With regard to lipids, *P. umbilicalis* extract (which is a marine plant extract) has the highest concentration of palmitic and oleic acids whereas *M. officinalis L*. and *E. officinalis* Gaertn extracts (terrestrial plants) contain linolenic and linoleic acids that were not detected in the extract of *P. umbilicalis*. *E. officinalis* Gaertn extract, the only fruit extract tested, showed the highest concentration of linoleic acid.

**Table 1:** Nutritional composition of the three plant extracts tested

	*P. umbilicalis*		*M. officinalis L*.		*E. officinalis* Gaertn		
Main components	Concentration % extract [% total fatty acids]	Daily dose per 500 mg extract	Concentration % extract [% total fatty acids]	Daily dose per 500 mg extract	Concentration % extract [% total fatty acids]	Daily dose per 125 mg/per 13 mg extract	Analysis method
Proteins	32.1	160.5	11.7	58.5	2.3	2.88/0.3	Internal method adapted from decree of 8 September 1977
Fibers (including polysaccharides)	40.3	201.5	31.6	158	19.3	24.1/2.5	AOAC 985.29
Carbohydrates	5.71	28.6	66	330	88.5	110.6/11.5	Internal method
Lipids	1.77	8.9	2.1	10.5	<0.5	<0.63/<0.08	Internal method adapted from decree of 8 September 1977
Saturated fatty acids	0.8 [42.5]	4	0.7 [35.3]	3.5	Traces [33.8]	Traces	Internal method
Monounsaturated fatty acids	0.4 [23.4]	2	0.3 [13.3]	1.5	Traces [21.3]	Traces	Internal method
Polyunsaturated fatty acids	0.6 [34.1]	3	1.1 [51.4]	5.5	Traces [44.9]	Traces	Internal method
Palmitic acid	[38.5]	—	[28.0]	—	[18.1]	—	Gas chromatography NFEN ISO 12966
Oleic acid (OA)	[15.3]	—	[4.8]	—	[18]	—	Gas chromatography NFEN ISO 12966
Eicosapentaenoic acid (EPA)	[9.9]	—	ND	—	ND	—	Gas chromatography NFEN ISO 12966
Arachidonic acid (AA)	[7.3]	—	ND	—	ND	—	Gas chromatography NFEN ISO 12966
Gamma‐linolenic acid (GLA)	[6.5]	—	ND	—	ND	—	Gas chromatography NFEN ISO 12966
Alpha‐linolenic acid (ALA)	ND	—	[31.7]	—	[14]	—	Gas chromatography NFEN ISO 12966
Linoleic acid (LA)	ND	—	[15.1]	—	[28.6]	—	Gas chromatography NFEN ISO 12966

ND: not detected (<0.1% of total fatty acids)

The extract of *M. officinalis L*. extract contained various polyphenol derivatives including rosmarinic acid, danshensu, 3′‐O‐(8″‐Z‐caffeoyl) rosmarinic acid, and flavones such as luteolin 3′‐O‐β‐D‐glucuronide (LC/MS analyses in negative ionization mode; internal data). The concentration of rosmarinic acid was 2.76 ± 0.05 mg per 100 mg of dried raw material (measured by HPLC).

In the extract of *E. officinalis* Gaertn, polyphenols including ellagic acid (0.10% w/w of dry material), gallic acid (0.11% w/w of dry material), methyl gallate (0.05% w/w of dry material), and ethyl gallate (0.09% w/w of dry material) were detected and quantified by HPLC (internal data). The other main components identified were β‐glucogallin (gallic acid derivative), 5‐O‐galloyl‐1,4‐galactarolactone isomers, chebulagic acid, and hirsutrin (not quantified). Quercetine was detected and quantified at 0.026% w/w of dry material (HPLC).

### 2.3 Dosage Information/Dosage Regimen

Plant extracts were dissolved in sterile water (vehicle, abbreviated as veh) and provided in special bottles (each with a metal bead avoiding waste of the plant extract solution used to fill it) for the mice to drink for 2 weeks (solutions changed every two days) at the following doses (quantity of plant extract powder/day/mouse): *P. umbilicalis* (Pum) 500 mg/day/mouse; *M. officinalis L*. (Mel) 500 mg/day/mouse); *E. officinalis* Gaertn: high dose (Eof‐H) 125 mg per day per mouse and low dose (ten times lower, Eof‐L) 13 mg per day per mouse.

Importantly, before treatment, water intake was measured for each group during two days and then the bottles were filled accordingly, to provide the doses reported above.

For the dose‐effect study, *E. officinalis* Gaertn was chosen, based on a clinical trial that evaluated the effect of 1000 mg of *E. officinalis* Gaertn. on endothelial function in patients with type 2 diabetes.^18^ This dose, equivalent to a dose of 250 mg kg^−1^ body weight per day calculated as the Human Equivalent Dose,^19^ proved most effective in that trial.

### 2.4 Taxonomic and Predicted Functional Analysis of the Gut Microbiota

**Figure 1:**
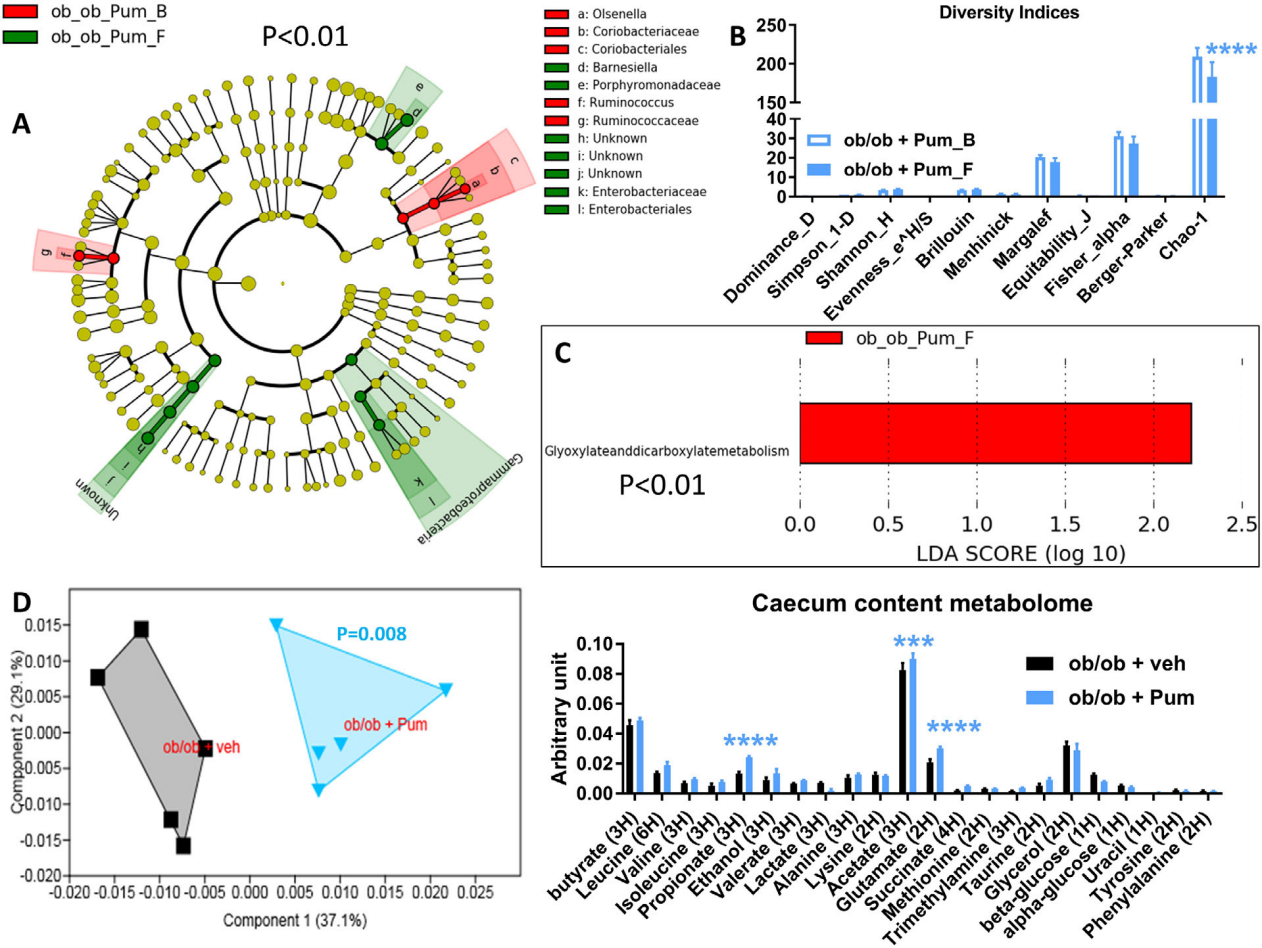
Baseline (B) to final (F) point comparative analysis by linear discriminant analysis (LDA) effect size (LEfSe) of the gut microbiota of ob/ob mice treated either with *Porphyra umbilicalis* (Pum) extract or vehicle for two weeks. *A*: Cladogram showing bacterial taxa significantly higher in the group of mice of the same color, in the fecal microbiota between the baseline (ob_ob_Pum_B/veh_B) and the final (ob_ob_Pum_F/veh_F) points (the cladogram shows the taxonomic levels represented by rings with phyla at the innermost and genera at the outermost ring and each circle is a bacterial member within that level). *B*: Indices of gut microbiota diversity. *C*: LDA score for predictive microbial pathway identified via PICRUSt.^22^ *D*: Principal component analysis (PCA) and histogram of the overall metabolites in the caecum content; *n* = 5. ***p < 0.001, ****p < 0.0001, two‐way ANOVA followed by a two‐stage linear step‐up procedure of Benjamini, Krieger and Yekutieli to correct for multiple comparisons by controlling the false discovery rate (<0.05); for (D) a one‐way PERMANOVA with a Bonferroni correction was applied. (Since the cladogram does not show the ob_ob_veh_B vs the ob_ob_veh_F groups, it means that the vehicle induced no significant changes in the gut microbiota of the control group at *p* < 0.01).

**Figure 2:**
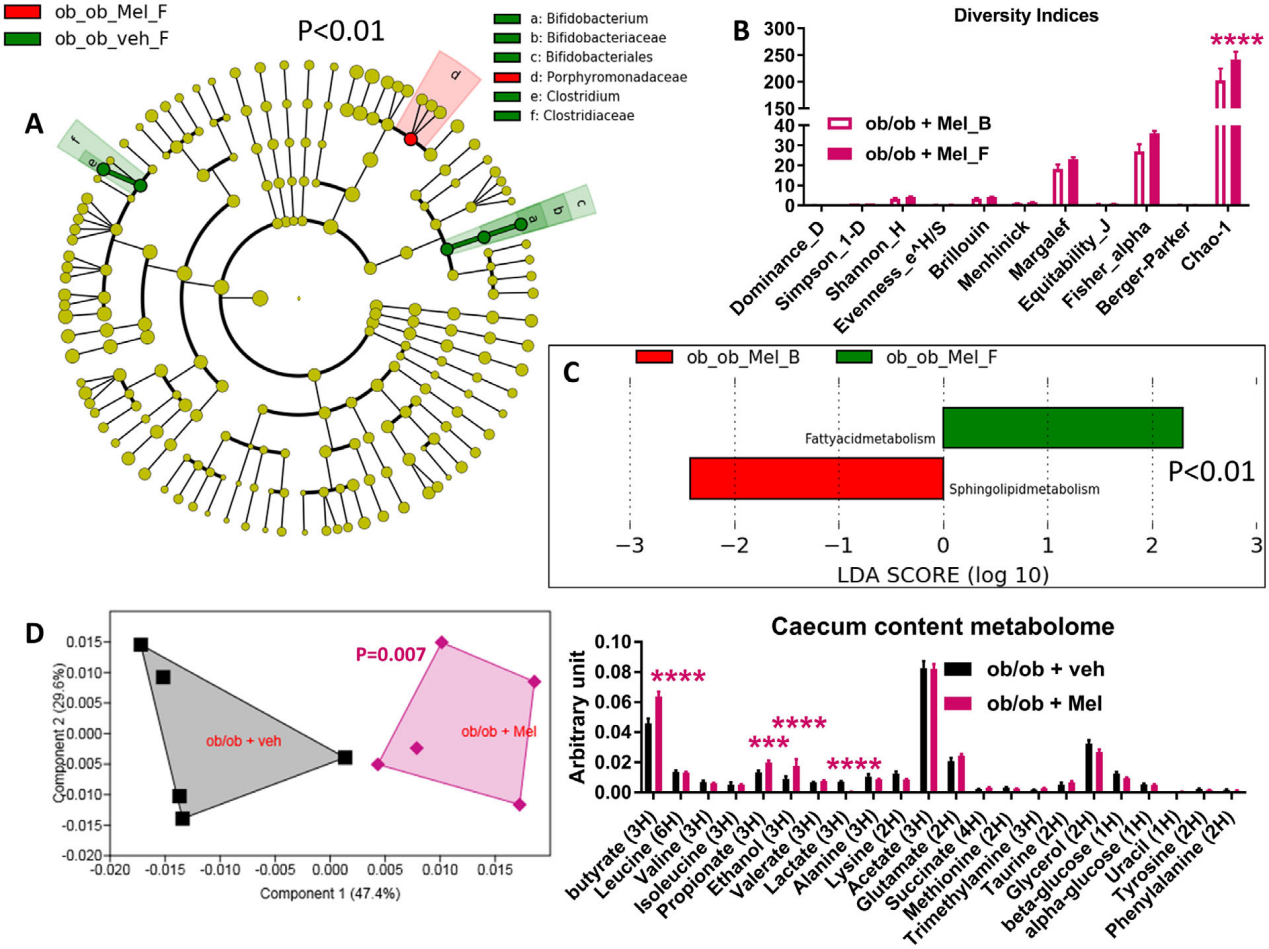
Baseline (B) to final (F) point comparative analysis by LEfSe of the gut microbiota of ob/ob mice treated with either *Melissa officinalis L*. (Mel) extract or vehicle for two weeks. *A*: Cladogram showing bacterial taxa significantly higher in the group of mice of the same color, in the fecal microbiota between the baseline (ob_ob_Mel_B/veh_B) and the final (ob_ob_Mel_F/veh_F) points. *B*: Indices of gut microbiota diversity. *C*: LDA score for predictive microbial pathway identified via PICRUSt.^22^ *D*: PCA and histogram of the total metabolites in the caecum content; *n* = 5. ***p < 0.001, ****p < 0.0001, two‐way ANOVA followed by a two‐stage linear step‐up procedure of Benjamini, Krieger and Yekutieli to correct for multiple comparisons by controlling the False Discovery Rate (<0.05), for (D) a one‐way PERMANOVA with a Bonferroni correction was applied. (Since the cladogram does not show the ob_ob_Mel_B and ob_ob_veh_B groups, it means that these groups are characterized by no higher bacterial taxon compared to the final point of the related group).

**Figure 3:**
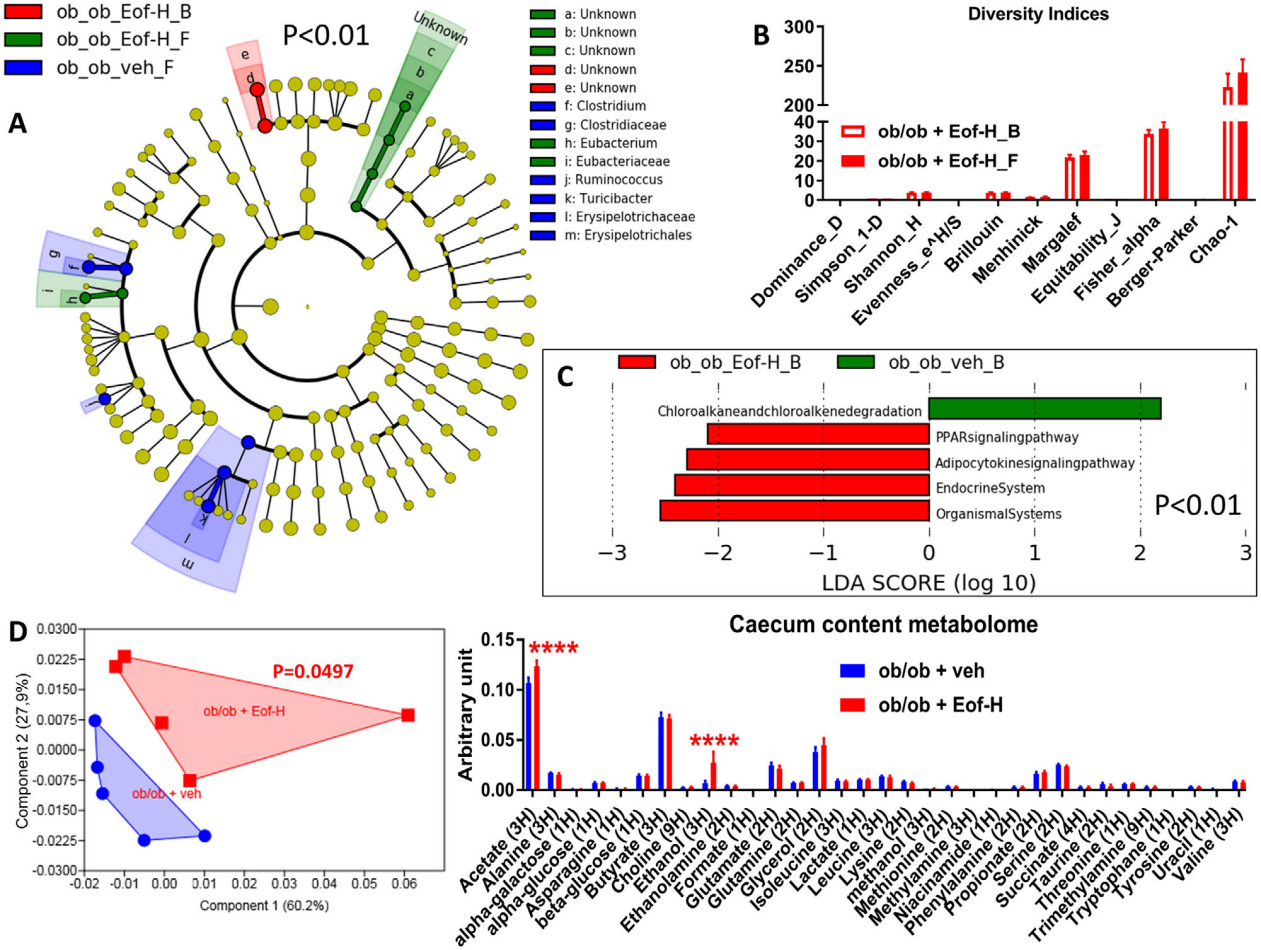
Baseline (B) to final (F) point comparative analysis by LEfSe of the gut microbiota of ob/ob mice treated with either a high dose of *Emblica officinalis* Gaertn (Eof‐H) extract or vehicle for two weeks. *A*: Cladogram showing bacterial taxa significantly higher in the group of mice of the same colour, in the fecal microbiota between the baseline (ob_ob_Eof‐H_B/veh_B) and the final (ob_ob_Eof‐H_F/veh_F) points. *B*: Indices of gut microbiota diversity. *C*: LDA score for predictive microbial pathway identified via PICRUSt.^22^ *D*: PCA and histogram of the total metabolites in the caecum content; *n* = 5. ****p < 0.0001, two‐way ANOVA followed by a two‐stage linear step‐up procedure of Benjamini, Krieger and Yekutieli to correct for multiple comparisons by controlling the False Discovery Rate (<0.05), for (D) a one‐way PERMANOVA with a Bonferroni correction was applied. (Since the cladogram does not show the ob_ob_veh_B group, it means that this group is characterized by no higher bacterial taxon compared to the final point of the related group).

**Figure 4:**
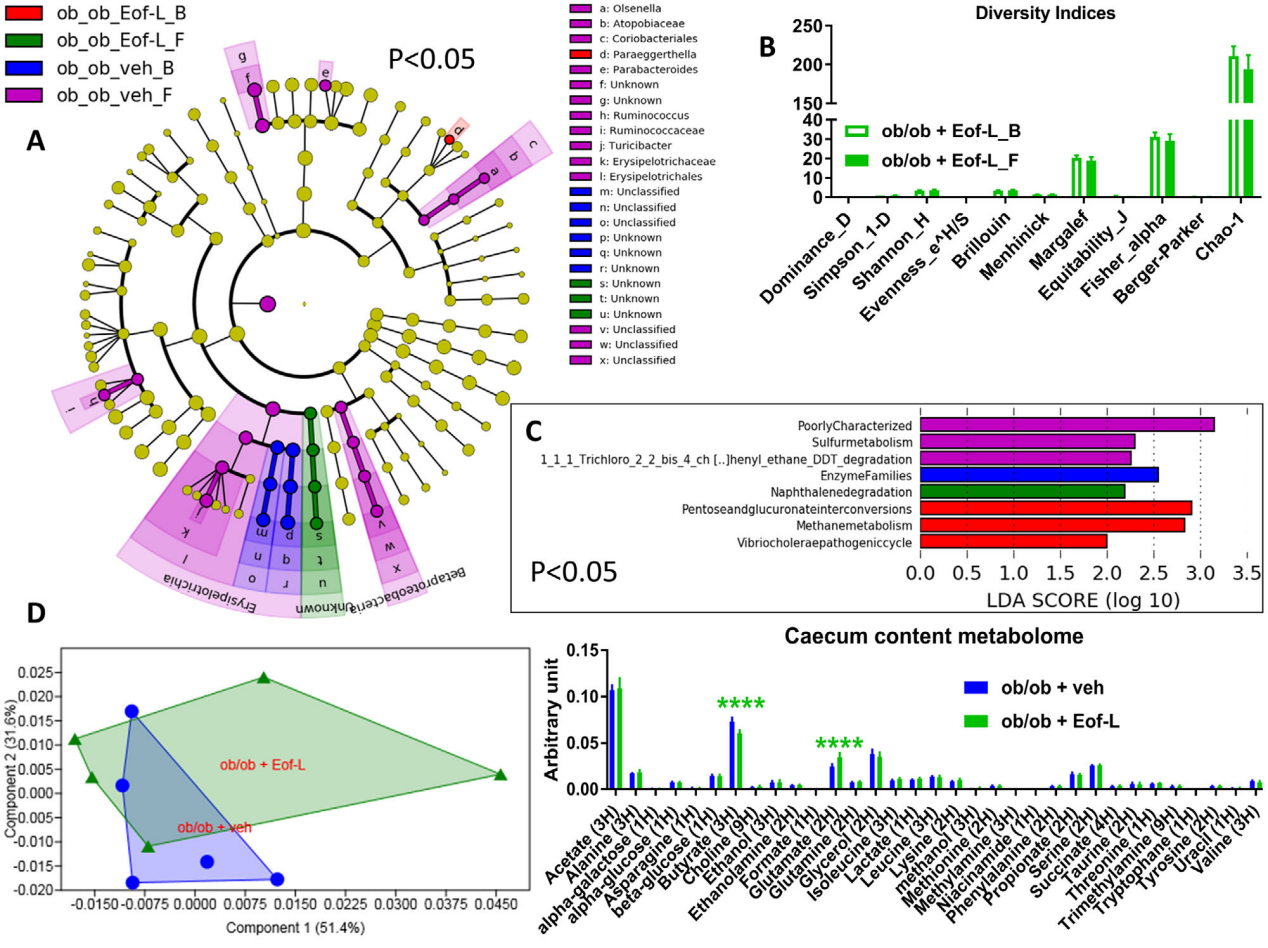
Baseline (B) to final (F) point comparative analysis by LEfSe of the gut microbiota of ob/ob mice treated with either a low dose of *Emblica officinalis* Gaertn (Eof‐L) extract or vehicle for two weeks. *A*: Cladogram showing bacterial taxa significantly higher in the group of mice of the same colour, in the fecal microbiota between the baseline (ob_ob_Eof‐L_B/veh_B) and the final (ob_ob_Eof‐L_F/veh_F) points. *B*: Indices of gut microbiota diversity. *C*: LDA score for predictive microbial pathway identified via PICRUSt.^22^ *D*: PCA and histogram of the overall metabolites in the caecum content; *n* = 5. ****p < 0.0001, two‐way ANOVA followed by a two‐stage linear step‐up procedure of Benjamini, Krieger, and Yekutieli to correct for multiple comparisons by controlling the False Discovery Rate (<0.05).

Total DNA was extracted from feces both at baseline and after treatment with the plant extracts as previously described.^20^ The 16S bacterial DNA V3‐V4 regions were targeted by 357wf‐785R primers and analyzed by MiSeq (RTLGenomics, http://rtlgenomics.com/, Texas, USA). An average of 21 600 sequences was generated per sample in the first study and of 23 234 in the second study. A complete description of the bioinformatic filters applied is available at http://www.rtlgenomics.com/docs/Data_Analysis_Methodology.pdf. The cladograms in **Figures** 1A,2A,3A,4A as well as LDA scores in Figures 1C,2C,3C,4C were drawn using the Huttenhower Galaxy web application (http://huttenhower.sph.harvard.edu/galaxy/) via the LEfSe algorithm.^21^ Diversity indices were calculated using the software Past 3.23 (Hammer, Ø., Harper, D.A.T., and P. D. Ryan, 2001. PAST: Paleontological Statistics Software Package for Education and Data Analysis. Palaeontologia Electronica 4(1): 9pp). The predictive functional analysis of the gut microbiota was performed via PICRUSt.^22^

### 2.5 Metabolomic Analysis of the Intestinal (Caecum) Content

The metabolomic (total metabolites) analysis of the caecum content was performed as previously described.^23^

### 2.6 Fed Blood Glucose Measurement, Oral Glucose‐Tolerance Test (OGTT), and Intraperitoneal Insulin‐Tolerance Test (IPITT)

Fed blood glucose was measured and OGTT and IPITT were performed at week 3, immediately after the two‐week treatment, as described elsewhere.^23^ IPITT was performed four days after OGTT and mice were fasted for 3 h and then injected with 5 U kg^−1^ insulin. The area under the curve (AUC) is shown in mmol/L x min as inset for OGTTs and calculated by the trapezoidal rule^24^ using GraphPad Prism version 7.00 for Windows Vista (GraphPad Software, San Diego, CA).

### 2.7 Biochemical Assays

Total plasma cholesterol, high‐ and low‐density lipoprotein (HDL and LDL, respectively), triacylglycerols (TG), and free fatty acids (FFA) were measured by multiplex assays on an ABX Pentra 400 machine by the Phenotypage ANEXPLO Platform (US06‐CREFRE). Plasma IL‐6 levels were measured by the Mouse IL‐6 Quantikine ELISA Kit (R&DSystems [a Bio‐techne brand], Lille, France), following the manufacturer's instructions.

### 2.8 RNA Extraction, Reverse Transcription, and qPCR

Total RNA was extracted from homogenized tissues (epididymal white adipose tissue or liver) using Tri Reagent solution (Euromedex, Souffelweyersheim, France) following the manufacturer's protocol. RNA purity was assessed using Nanodrop (Thermo, Evry, France). For reverse transcription, 1 µg of RNA was used with 1 U of M‐MLV Reverse transcriptase (Thermo), 15 ng random hexamers, 10 mm DTT, and 1 mm dNTPs. After 1 h at 37 °C, reverse transcriptase was inactivated by heating for 10 min at 65 °C. cDNAs were diluted 1:5 with ultrapure water. For qPCR reactions, 2.5 µL cDNA were mixed with 6.25 µL of Taqman UNIV PCR Master mix 2X, 0.625 µL Taqman assay 20X (Thermo), and 3.125 µL of ultrapure water. Amplification was performed in a Stratagene Mx 3005P thermocycler (Agilent, Les Ulis, France) using the following temperature conditions: 2 min at 50 °C, 10 min at 95 °C, and 40 cycles of alternance of 15 s at 95 °C per 1 min at 60 °C. For each condition, expression was quantified in duplicate and 18S rRNA was used as the endogenous control in the comparative cycle threshold (CT) method. The Taqman assay identification number for each couple of primers used to study the related gene is available upon request.

### 2.9 Statistical Analysis

The results are presented as mean ± SEM. *n* = 5. Statistical analyses were performed by two‐way ANOVA followed by a two‐stage linear step‐up procedure of Benjamini, Krieger, and Yekutieli to correct for multiple comparisons by controlling the False Discovery Rate (<0.05) or Kruskal–Wallis test plus a two‐stage step‐up method of Benjamini, Krieger and Yekutieli correction for multiple comparisons by controlling the False Discovery Rate (<0.05) or Mann–Whitney test, as indicated in the figure legend, by using GraphPad Prism version 7.00 for Windows Vista (GraphPad Software, San Diego, CA). Significant values were considered starting at *p* < 0.05 or as reported after corrections. For the taxonomical and predictive functional analysis of gut microbiota significant values were considered starting at *p* < 0.01 (Figures 1, 2, 3). For the correlation between IL‐6 and the order Clostridiales the P value was corrected according to the Benjamini–Hochberg correction for multiple comparisons, with a false discovery rate <0.05, *n* = 15 (all mice from the first study). Principal Component Analysis graphs were drawn and related statistical analyses were performed by using Past 3.23 and one‐way PERMANOVA analysis with Bonferroni's correction.

## 3 Results

### 3.1 Analysis of Gut Microbiota in ob/ob Mice after a Two‐Week Treatment with Plant Extracts

The aim of this study was to evaluate the putative prebiotic properties of plant extracts on gut microbiota during a short treatment period of two weeks. As our objective was to investigate the impact of these extracts on a dysbiotic gut microbiota, the experiments were conducted in ob/ob mice, a known murine model of gut microbiota dysbiosis.^12^ We compared the gut microbiota before (*baseline point*, B) and after the treatment (*final point*, F), for each group of mice. To strengthen our results and avoid their over‐interpretation, we also performed the analysis described above on a control group of mice (one group per study) treated with the vehicle (sterile water) in which the plant extracts were dissolved.

Treatment with *Porphyra umbilicalis* (Pum) induced a significant increase in bacteria belonging to the phylum Gammaproteobacteria and to the families *Enterobacteriaceae* and *Porphyromonadaceae* as well as the genus *Barnesiella* (the two last groups belonging to phylum Bacteroidetes); by contrast, the Pum treatment appeared to reduce the abundance of the bacterial order Coriobacteriales and its component taxon Olsenella and the bacterial family *Ruminococcaceae* (Figure 1A); in terms of overall microbial diversity, treatment with Pum decreased the Chao‐1 diversity index, which indicates the diversity related to rare species (Figure 1B). Then, we analysed the microbial pathways that could be affected by Pum treatment by performing a PICRUSt‐based predictive analysis of the microbiome.^22^ The analysis showed that Pum treatment increased the microbial pathway related to glyoxylate and dicarboxylate metabolism (Figure 1C).

Mice receiving *Melissa officinalis L*. (Mel) extract showed a significant increase in the bacterial family Porphyromonadaceae (Figure 2A); in terms of overall microbial diversity, treatment with Mel increased the Chao‐1 diversity index (Figure 2B). With respect to the predicted microbiome, treatment with Mel increased the microbial pathway related to fatty acid metabolism and appeared to reduce that related to sphingolipid metabolism, found to be enriched at baseline (Figure 2C).

Treatment with the high dose of *Emblica officinalis* Gaertn (Eof‐H) increased the abundance of the genus *Eubacterium* belonging to the family *Eubacteriaceae* (belonging to the Firmicutes phylum, Figure 3A) without affecting either the overall microbial diversity (Figure 3B) or the predicted microbiome (Figure 3C). By contrast, the changes induced by the low dose of *E. officinalis* Gaertn (Eof‐L) were modest and the taxa implicated were not identifiable (unknown taxa), though at baseline mice displayed a higher abundance of the genus *Paraeggerthella*, thus likely decreased by Eof‐L (Figure 4A). These data were associated with no change in overall microbial diversity (Figure 4B) but with an increased naphthalene degradation microbial pathway (Figure 4C).

Overall, these data show that the plant extracts tested are capable of changing the dysbiotic gut microbiota of ob/ob mice over a short period of two weeks, Eof‐H being more effective than Eof‐L.

### 3.2 Metabolomic Analysis of the Intestinal (Caecum) Content of ob/ob Mice Treated with Plant Extracts for Two Weeks

To measure the metabolization of plant extracts by gut bacteria in terms of production of both short‐chain fatty acids (SCFAs) (key molecules of microbial origin involved in the modulation of host metabolism^25^, ^26^, ^27^) and other metabolites, we performed a metabolomic analysis of the intestinal (caecum) content.^28^, ^29^, ^30^ The overall caecal metabolomic profiles of control mice differed to a highly significant extent from those of mice treated with Pum or Mel extracts, as evidenced by an independent cluster for each (Figures 1D and  2D, left panel). In detail, mice treated with Pum extract showed significant higher levels of propionate, acetate, and glutamate (Figure 1D, right panel); mice treated with Mel extract showed significantly higher levels of butyrate, propionate, and ethanol and a significantly lower level of lactate (Figure 2D, right panel).

Mice treated with Eof‐H showed an overall caecal metabolomic profile significantly dissimilar from that of the control group manifesting a significantly higher level in both acetate and ethanol (Figure 3D, left and right panel, respectively); by contrast, mice treated with Eof‐L showed an overall caecum content profile similar to that of control mice, despite a significantly lower level in butyrate and a significantly higher level in glutamate (Figure 4D, left and right panel, respectively).

Overall, these data show that a two‐week treatment with *P. umbilicalis* and *M. officinalis L*. extracts affects the caecum metabolome in a murine model of genetically‐induced obesity.

### 3.3 Analysis of Markers of Lipid Metabolism in the Plasma of ob/ob Mice Treated with Plant Extracts for Two Weeks

Since we observed changes in both the gut microbiota and the caecum metabolome and it is known that gut microbiota can affect lipid homeostasis,^26^, ^31^, ^32^, ^33^ we therefore analyzed certain key markers of lipid metabolism. In the first study, ob/ob mice treated with Pum extract had significantly lower plasma TG levels compared to control mice (**Table** 2); by contrast, in the second study, ob/ob mice treated with Eof‐L showed significantly higher plasma FFA levels compared to control mice. Overall, these data show that a short treatment of two weeks with *P. umbilicalis* extracts and a low dose of *E. officinalis* Gaertn extract can affect key markers of lipid metabolism in a murine model of genetically‐induced obesity.

**Table 2:** Effects of the treatment with plant extracts on the lipid metabolism in the plasma of ob/ob mice. Assay of total cholesterol, cholesterol HDL, cholesterol LDL, triacylglycerols (TG), and free fatty acids (FFA) in the plasma

Study	Treatment	Tot chol [mg dL^−1^]	HDL [mg dL^−1^]	LDL [mg dL^−1^]	TG [mg dL^−1^]	FFA [mg dL^−1^]
1	Veh	5.7 ± 0.2	2.18 ± 0.12	0.71 ± 0.04	1.09 ± 0.04	0.62 ± 0.15
	Pum	5.4 ± 0.2	2.30 ± 0.04	0.62 ± 0.05	0.80 ± 0.02**	0.74 ± 0.06
	Mel	4.9 ± 0.4	2.11 ± 0.11	0.58 ± 0.06	1.12 ± 0.15	0.96 ± 0.14
2	Veh	5.97 ± 0.27	2.31 ± 0.08	0.51 ± 0.03	1.16 ± 0.11	0.49 ± 0.05
	Eof‐H	5.73 ± 0.34	2.36 ± 0.08	0.49 ± 0.05	1.02 ± 0.05	0.56 ± 0.03
	Eof‐L	5.28 ± 0.84	1.98 ± 0.35	0.51 ± 0.08	1.3 ± 0.2	0.69 ± 0.05*

^*^ *p* < 0.05

^**^ *p* < 0.01, Mann‐Whitney test vs the related control group treated with sterile water (vehicle, veh). Pum: *Porphyra umbilicalis* extract; Mel: *Melissa officinalis L*. extract; Eof‐H: high‐dose *Emblica officinalis* Gaertn extract; Eof‐L: low‐dose *Emblica officinalis* Gaertn extract; *n* = 5 per group.

### 3.4 Analysis of Markers of Glucose Metabolism and Survey of Body Weight in ob/ob Mice Treated with Plant Extracts for Two Weeks

**Figure 5:**
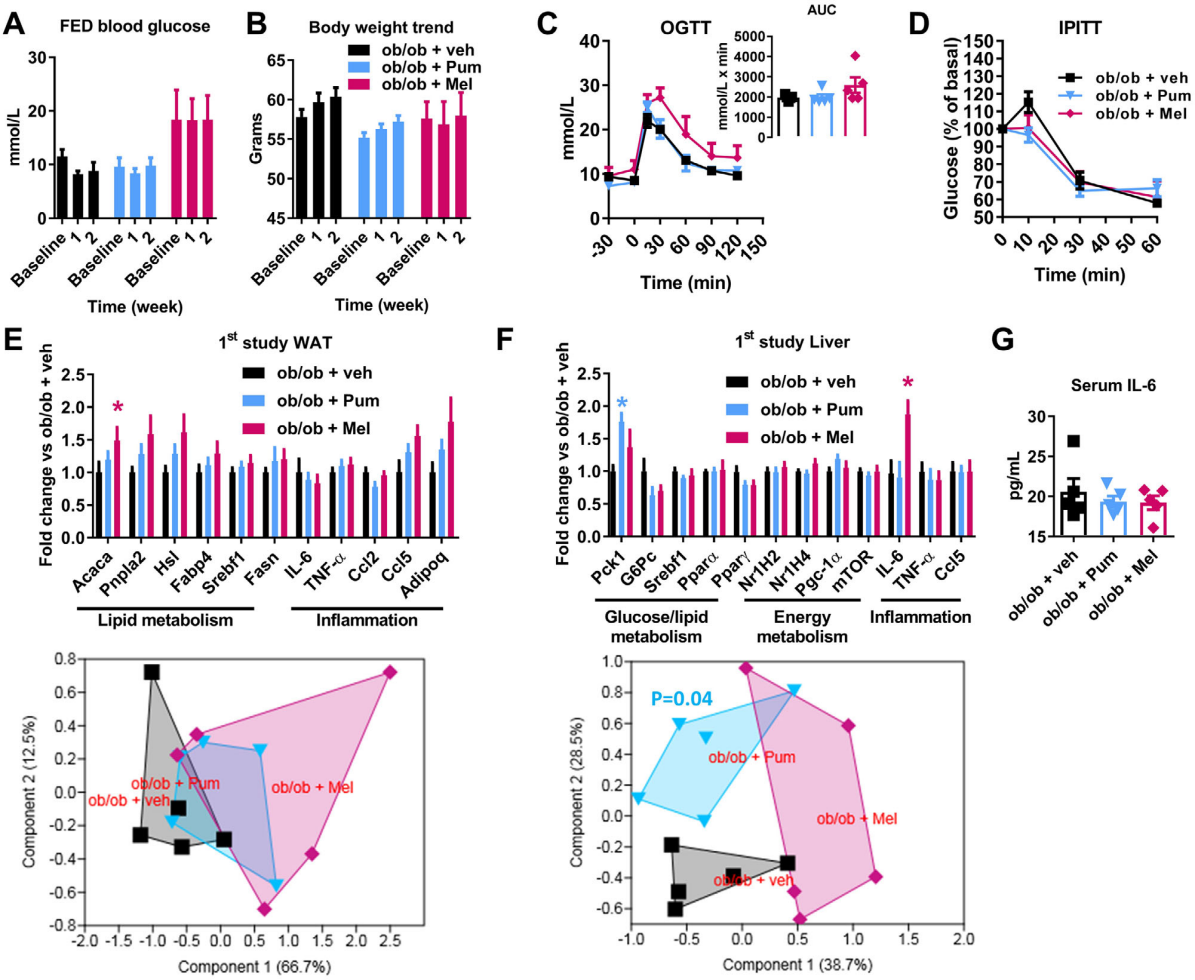
Markers of glucose homeostasis and survey of body weight in ob/ob mice following a two‐week treatment with plant extract from *Porphyra umbilicalis* (Pum) or *Melissa officinalis L*. (Mel) or vehicle (veh). Kinetic analysis of *A*: fed blood glucose and *B*: body weight during the two weeks of treatment. *C*: oral glucose‐tolerance test (OGTT) with AUC (area under the curve) as inset. *D*: intraperitoneal insulin‐tolerance test (IPITT). Gene expression analysis of white adipose tissue (*E*) and liver (*F*) and related PCA as subpanel. *G*: Serum IL‐6 levels. Data are presented as mean ± SEM. *n* = 5; *p < 0.05, Kruskal–Wallis test (*E, F*) plus two‐stage step‐up method of Benjamini, Krieger and Yekutieli correction for multiple comparisons by controlling the false discovery rate (FDR < 0.05) vs control group ob/ob + veh or 1‐PERMANOVA (*F*, lower panel) with Bonferroni's corrections.

Given the link between lipid and glucose metabolism and gut microbiota activity,^32^, ^34^ we analyzed markers of glucose metabolism in vivo and also monitored body weight. Treatment with Pum and Mel extracts did not significantly affect neither fed blood glucose nor body weight (**Figure** 5A,B, respectively). Then, to determine whether these plant extracts may affect blood glucose on a dynamic basis, we performed an OGTT and an IPITT. No significant changes were observed irrespective of the parameter and the group (Figure 5C,D).

**Figure 6:**
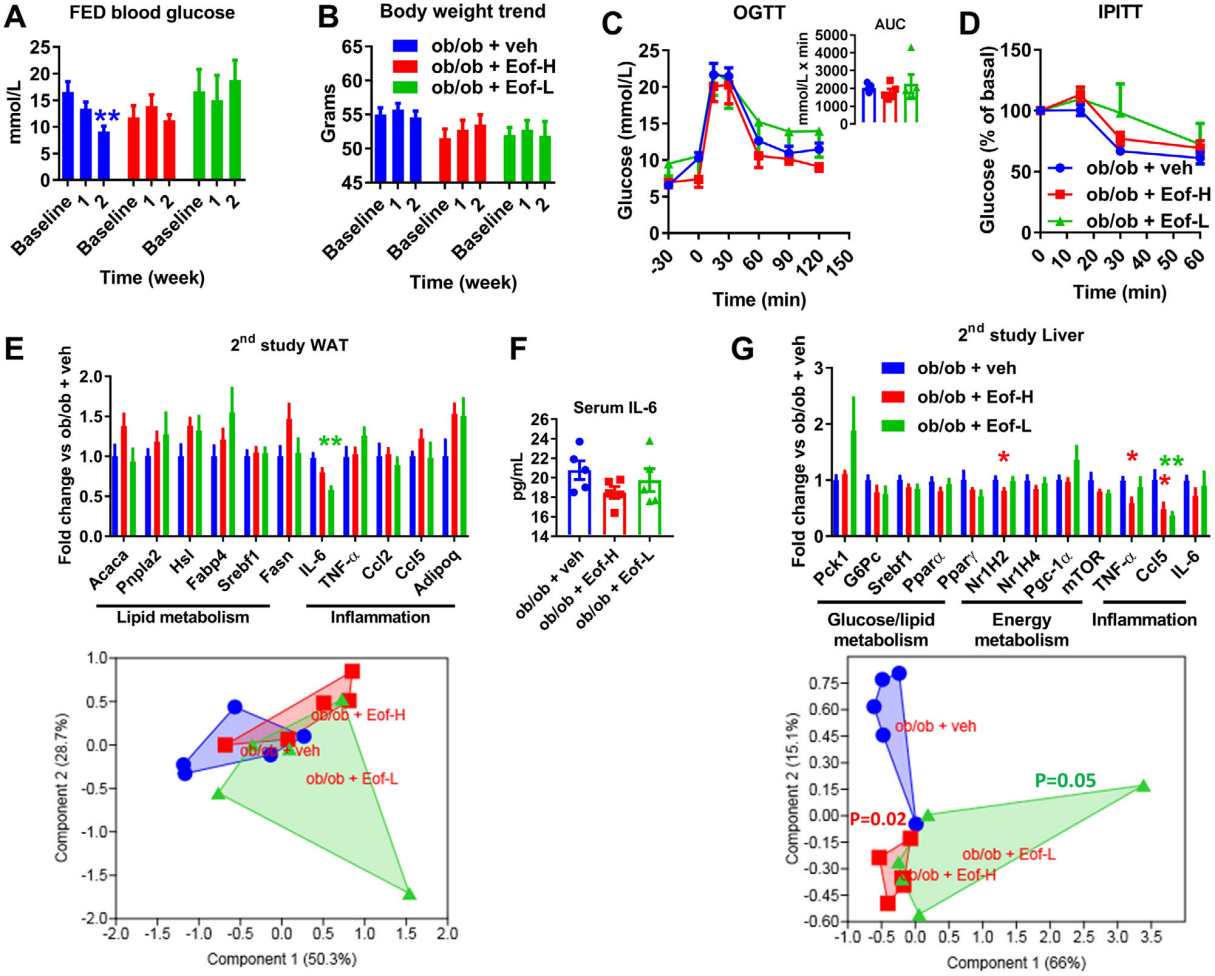
Markers of glucose homeostasis and survey of body weight in ob/ob mice following a two‐week treatment with plant extract from *Emblica officinalis* Gaertn high‐dose (Eof‐H) or low dose (Eof‐L) or vehicle (veh). Kinetic analysis of *A*: fed blood glucose and *B*: body weight during the two weeks of treatment. *C*: oral glucose‐tolerance test (OGTT) with AUC (area under the curve) as inset. *D*: intraperitoneal insulin‐tolerance test (IPITT). Gene expression analysis of white adipose tissue (*E*) and liver (*G*) and related PCA as subpanel. *F*: Serum IL‐6 levels. Data are presented as mean ± SEM. *n* = 5; *p < 0.05, **p < 0.01, Kruskal‐Wallis test (*A, E, G*) plus two‐stage step‐up method of Benjamini, Krieger and Yekutieli correction for multiple comparisons by controlling the false discovery rate (FDR < 0.05) vs control group ob/ob + veh or 1‐PERMANOVA (*G*, lower panel) with Bonferroni's corrections.

Treatment with Eof‐H and Eof‐L extracts did not significantly affect neither fed blood glucose nor body weight; the control group showed a significant reduction of the body weight over two weeks (**Figure** 6A,B). Both OGTT and IPITT were not significantly affected irrespective of the group (Figure 6C,D).

Overall, these data show that, in a murine model of genetically‐induced obesity, a two‐week treatment with the tested plant extracts did not significantly affect markers of glucose metabolism, despite the changes observed in gut microbiota, caecum metabolome, and lipid metabolism.

### 3.5 Analysis of Gene Expression in White Adipose Tissue and Liver of ob/ob Mice Treated with Plant Extracts for Two Weeks

A targeted analysis of the expression of genes related to lipid, glucose, and energy metabolism as well as inflammation was performed in white adipose tissue (WAT) and liver. Mice treated with Mel extract showed a significantly higher expression of the gene *Acaca* (acetyl‐CoA carboxylase alpha, related to lipogenesis) in the WAT (Figure 5E, upper panel). However, the expression profile for all genes analyzed in the WAT was not dissimilar to that of control mice, whatever the treatment (Figure 5E, lower panel). By contrast, in the liver, a significantly higher expression of the genes *Pck1* (phosphoenolpyruvate carboxykinase 1, related to glucose metabolism) and *IL‐6* (interleukin‐6, related to inflammation) was observed, in mice treated with Pum and Mel extracts, respectively (Figure 5F, upper panel). Moreover, mice treated with Pum, but not those treated with Mel, displayed a significantly different expression profile for all hepatic genes analysed compared to control mice (Figure 5F, lower panel). The change in the hepatic expression of gene *IL‐6* was not associated with a change in IL‐6 plasma levels, in any group (Figure 5G).

Eof‐H extract did not significantly affect WAT gene expression whereas mice treated with Eof‐L extract showed a significantly lower expression of the gene *IL‐6* in the WAT (Figure 6E, upper panel), which was not associated with a change in IL‐6 plasma levels, whatever the group (Figure 6F). The expression profile for all genes analysed in the WAT was not dissimilar to that of control mice, whatever the treatment (Figure 6E, lower panel).

In the liver, a significantly lower expression was observed for genes *Nr1H2* (nuclear receptor subfamily 1, group H, member 2, related to energy metabolism), *TNF‐α*, and *Ccl5* (tumor necrosis factor‐ *α* and chemokine (C‐C motif) ligand 5, respectively, both related to inflammation), in mice treated with Eof‐H; by contrast, mice treated with Eof‐L only showed a significantly lower expression in gene *Ccl5* expression (Figure 6G, upper panel). Interestingly, both mice treated with Eof‐H and those treated with Eof‐L displayed a significantly different expression profile for all hepatic genes analysed compared to control mice (Figure 6G, lower panel).

Overall, these results suggest a plant extract‐dependent and tissue‐specific effect on the expression of genes analysed in the WAT and the liver of ob/ob mice.

We also investigated whether the expression of the genes significantly modulated in both studies and tissues might be correlated with specific bacterial taxa as well as microbial pathways of the gut microbiota together with caecum metabolites and IL‐6 plasma levels. We did not identify any significant correlation between the expression of genes in the WAT and bacterial taxa in either study (data not shown). By contrast, in the first study, the hepatic expression of *IL‐6* (but not *IL‐6* plasma levels) was found to be significantly (adjusted *p* = 0.0015) and positively (*r* = 0.80, Spearman correlation) correlated with the bacterial order Clostridiales.

## 4 Discussion

The aim of this study was to evaluate whether extracts of three plants *Porphyra umbilicalis*, *Melissa officinalis L*., and *Emblica officinalis* Gaertn might have prebiotic properties when administered for two weeks to ob/ob mice. Our criteria to define prebiotic properties were: i) the impact on gut microbiota, ii) generation of SCFAs, and iii) improvement in lipid and/or glucose metabolism. Since in current medical practice prebiotics are proposed to patients experiencing gut discomfort, we chose ob/ob mice, a known model of metabolic diseases and gut microbiota dysbiosis.^12^ Targeting the gut microbiota of ob/ob mice was already proven to be effective in ameliorating glucose metabolism,^35^ supporting our rationale. Moreover, prebiotics are usually administered for a long period (at least four weeks) and at high doses (up to 10 g per day per patient). Thus, some patients may experience undesirable effects such as gut ballooning and/or increased flatulence^7^, ^8^ (parameters that could not be taken into account in our study), due to fermentation of prebiotics by gut bacteria.^9^ Based on these evidences, we opted for a short period of two weeks and the use of polyphenol extracts. Importantly, intestinal discomfort (ballooning and flatulence) has not been reported in clinical studies after the administration of such extracts.^36^, ^37^, ^38^

The modifications in the gut microbiota induced by Pum and Mel extracts were associated with a significant shift of the overall metabolome of the caecum content. In particular, mice treated with Pum and those treated with Mel displayed higher caecal levels of propionate than control mice. It is also noteworthy that Pum induced a substantial reduction in plasma TG, which could be mechanistically linked to the observed increase in propionate, as previously reported.^39^ The increase in propionate and reduction in TG were associated with a change in hepatic gene expression but not with a modulation of glucose metabolism. Certain prebiotics, such as oligofructoses, have been shown to be capable of increasing glucose‐tolerance, improving intestinal physiology and reducing fat‐mass and inflammation in the same murine model as that we used.^40^ However, in that study, the ob/ob mice were younger (10 weeks old) and the treatment with oligofructoses^41^ (added to a control diet in a proportion of 9:1 [weight of control diet:weight of fibers]) was longer (5 weeks) than the protocol we employed. These differences could provide an explanation for the inconsistency between these previously reported data and our findings.

With regard to the modulation of gut microbiota, treatment with Pum extract indirectly reduced the abundance of the genus *Ruminococcus*, found to be higher at baseline. The genus *Ruminococcus* was positively associated with a better lipid utilization in mice, via the production of the bacteriocin Albusin B, a toxin capable of enhancing lipid utilization in BALB/c mice.^42^ These data are in contrast with ours since we show that reduced plasma TG are instead associated with reduced levels of *Ruminococcus*. This discrepancy suggests that bacteria belonging to the *Ruminococcus* genus may have different metabolic effects depending on both the pathophysiology and the genetic background of the animal model used (BALB/c vs C57Bl/6 ob/ob mice).

Modifications of the gut microbiota induced by Eof‐H were associated with a significant shift of the overall metabolome of the caecum content. Specifically, mice treated with Eof‐H displayed higher levels in both acetate and ethanol. These changes were associated with a modulation of the gene expression in the liver. In mice treated with Eof‐L we observed a modest impact on the gut microbiota (related to unknown taxa) and the predicted microbial pathways. Moreover, this treatment did not induce a significant shift of the overall metabolome, despite the observed lower levels of butyrate and higher levels of glutamate. The butyrate levels were also modulated by Mel in the first study, though toward higher levels. The effective impact of butyrate could be controversial since its increase could favor infections by certain pathogenic *E. coli* such as the strain O157:H7, as shown by Zumbrun et al.^43^ Modulations of butyrate levels may therefore represent either a positive or a negative outcome, depending on a very specific context.^44^

In terms of metabolic modulations, mice treated with Eof‐L exhibited higher plasma levels of FFA. This finding could reflect either increased lipolysis or reduced lipid storage into the adipose tissue^32^ and was associated with a lower *IL‐6* gene expression in the WAT. Increased FFA plasma levels are generally associated with a detrimental metabolic condition. However, elevated FFA plasma levels have been demonstrated in axenic mice fed a high‐fat diet, which are known to resist diet‐induced obesity.^32^ Thus, based on this study reported by Bäckhed et al., the increase in FFA we observed in mice treated with Eof‐L could be interpreted as a reduction in energy storage in the WAT of ob/ob mice, which could represent a favorable metabolic condition.

In conclusion, a two‐week treatment with *Porphyra umbilicalis*, *Melissa officinalis L*., and *E. officinalis* Gaertn extracts can significantly affect a dysbiotic gut microbiota, caecum metabolome, and markers of lipid metabolism in ob/ob mice. We also observed a change in the expression of certain genes involved in glucose, lipid, energy metabolism, as well as inflammation in both WAT and liver. The extent of these changes was dependent on both the nature of the plant‐extract administered and the dose. With regard to the dose, mice cohousing during treatment may have induced variation in the dosing of each animal. However, we have not observed aberrant intragroup variances for whatever the studied parameter, suggesting that the putative variation in the consumption occurred to a little extent.

Nevertheless, these changes were associated neither with modulations of body weight nor glucose metabolism and in general cannot be identified as metabolic improvements. Thus, in terms of validation of the tested plant extracts as prebiotics, apart from the changes induced in the gut microbiota, we face a dichotomy between the effects observed on gut microbiota and lipid metabolism vs those observed on glucose metabolism. Despite our intent was not to compare plant extracts to each other but rather to appreciate metabolic intra‐group effects, baseline difference in body weight among the groups of mice might be a limitation to further metabolic interpretation. Moreover, for the translational relevance of our results, more data on patients are required to assess whether both a limited concentration of prebiotics and a reduced time of treatment (2 weeks) could be effective and limit side‐effects.

Our data may stimulate a debate on how any substrate with putative prebiotic properties should be validated, and in particular whether this validation should target a specific parameter or a wider panel of metabolic parameters, regardless of changes in the gut microbiota and/or microbiome.

## Conflict of Interest

Groupe PiLeJe (Saint‐Laurent‐des‐Autels, France) funded M.S. for this study. A.B. was formerly employed by PiLeJe. There are no patents pending.
